# Pembrolizumab-Induced Eruptive Keratoacanthomas and Lichen Planus in a Lung Cancer Patient

**DOI:** 10.7759/cureus.43402

**Published:** 2023-08-13

**Authors:** Yazmeen Tembunde, Marthe N Dika

**Affiliations:** 1 Dermatology, University of Maryland School of Medicine, Baltimore, USA; 2 Dermatology, M. Dika Dermatology, Burlington, USA

**Keywords:** methotrexate, lung cancer, keratoacanthoma, lichen planus, pembrolizumab

## Abstract

Reports of pembrolizumab-induced lichen planus and eruptive keratoacanthomas are limited in the literature. Here, we describe the unique concurrence of both lichen planus and eruptive keratoacanthomas in a patient who received pembrolizumab for non-small cell lung cancer (NSCLC). Although several therapies have been proposed, we show that pembrolizumab-induced lichen planus and keratoacanthomas can be controlled with the conservative management of topical corticosteroids and intralesional corticosteroids, respectively, allowing patients to continue pembrolizumab therapy.

## Introduction

Pembrolizumab, a monoclonal antibody directed against the programmed cell death 1 protein (PD-1), is commonly used to treat malignancies such as melanoma and non-small-cell lung cancer (NSCLC) [1,2]. Its increasing popularity and use have revealed various dermatologic adverse events (dAEs) [1,3]. Pembrolizumab-induced lichen planus and eruptive keratoacanthomas have been previously described in the literature [2]; however, documented cases are limited. More case reports are needed to demonstrate the full range of dAEs. We report the unique concurrence of both lichen planus and eruptive keratoacanthomas in a patient who received pembrolizumab for NSCLC.

## Case presentation

The patient is a 67-year-old female who presented with a concern for a pruritic rash on her upper back. She stated that the rash started a week after beginning pembrolizumab for NSCLC seven months ago. The patient was receiving pembrolizumab 200 mg intravenous infusions every three weeks. The rash was intermittent, typically present for one to two weeks before resolving spontaneously. She didn’t notice a temporal relationship between the infusions and rash reappearance. She was previously prescribed triamcinolone 0.5% cream and mometasone 0.1% cream for the rash, but these treatments only provided minimal relief of symptoms. She also stated that around the time of noticing this rash, she began experiencing a sore over her right anterior shin that was painful to the touch and two similar sores on her anterior left shin. The patient reported that these sores have been constant and denied any drainage from the areas.

**Figure 1:**
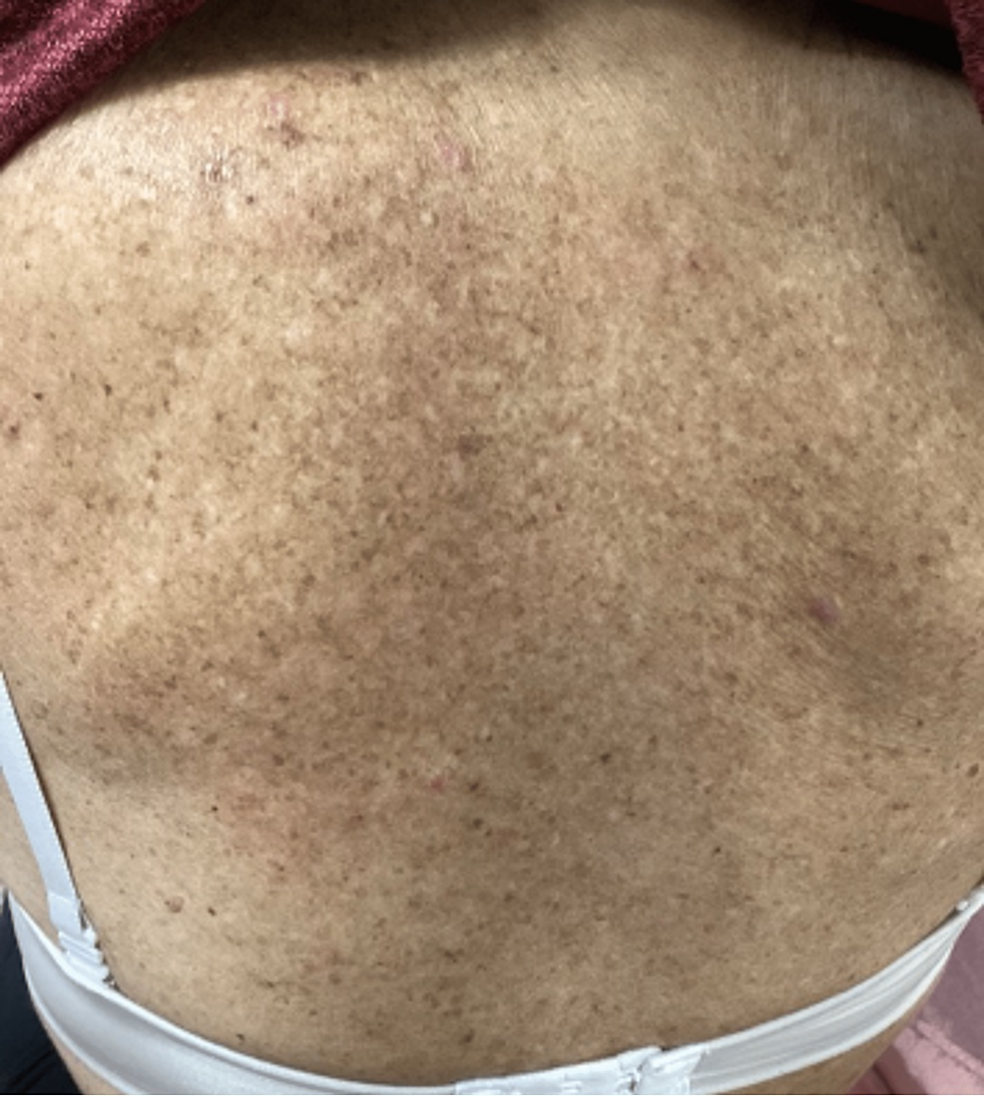
Upper back rash, prior to treatment

Physical examination of the upper back showed purple to violaceous, polygonal, shiny, flat-topped firm papules and plaques with evidence of Wickham striae (Figure 1). This rash was diagnosed as a lichenoid drug eruption, secondary to pembrolizumab, and for this rash, she was prescribed triamcinolone acetonide 0.1 % cream to be applied on her back twice a day for 30 days.

**Figure 2:**
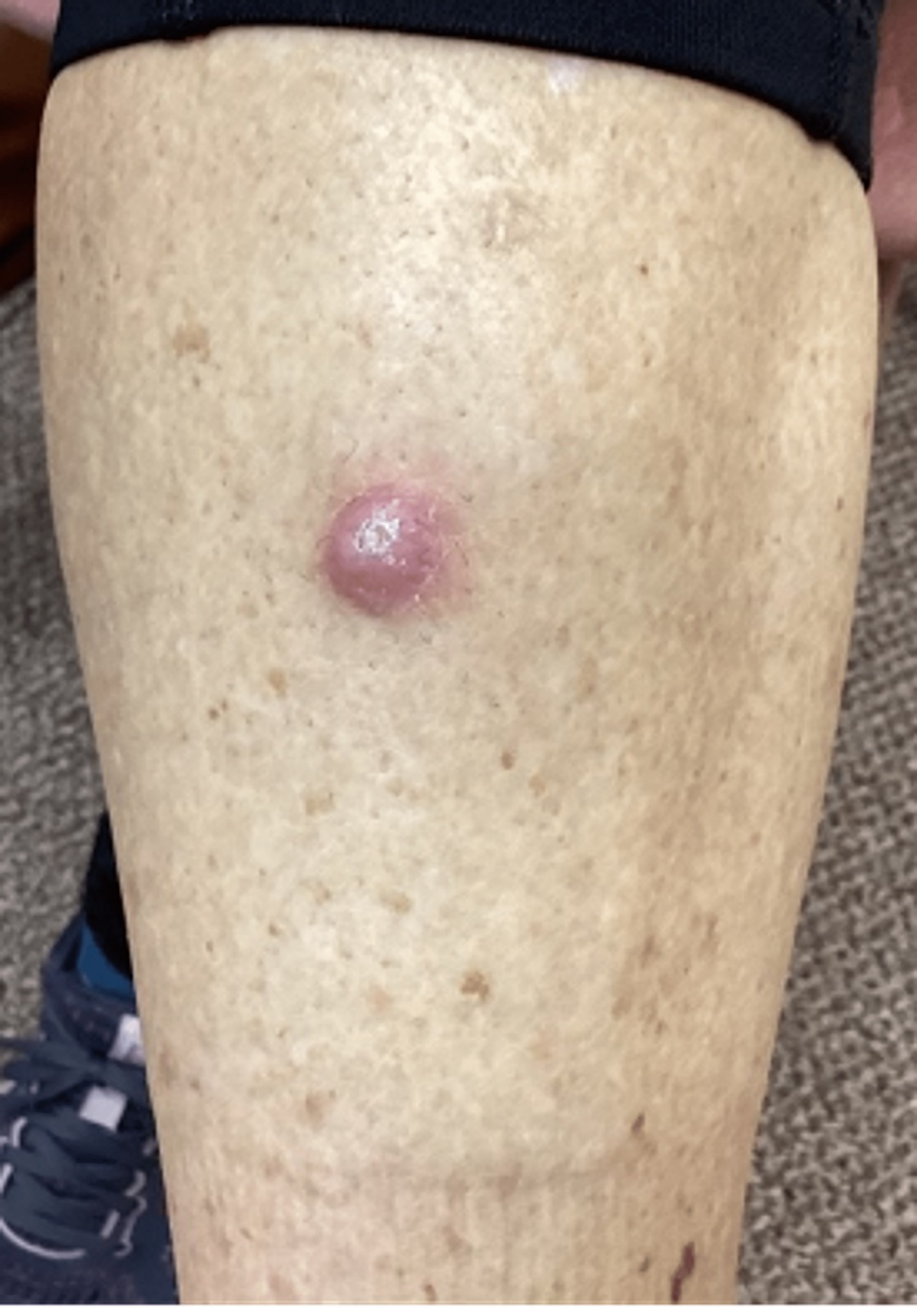
Right anterior lower leg lesion prior to biopsy and treatment

**Figure 3:**
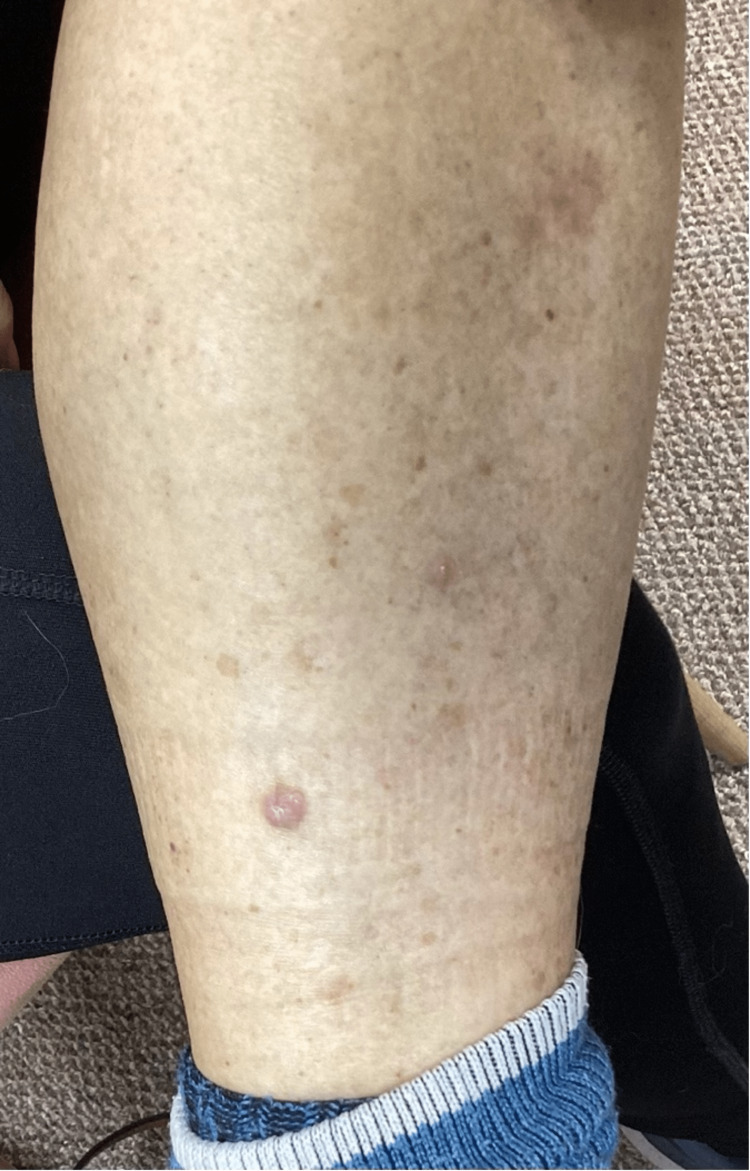
Left lower leg lesions, prior to treatment

Examination of the lower extremities revealed a 1.3 cm erythematous hyperkeratotic papule on her proximal right anterior lower leg (Figure 2) and smaller, similar-looking lesions on her left lower leg (Figure 3).

**Figure 4:**
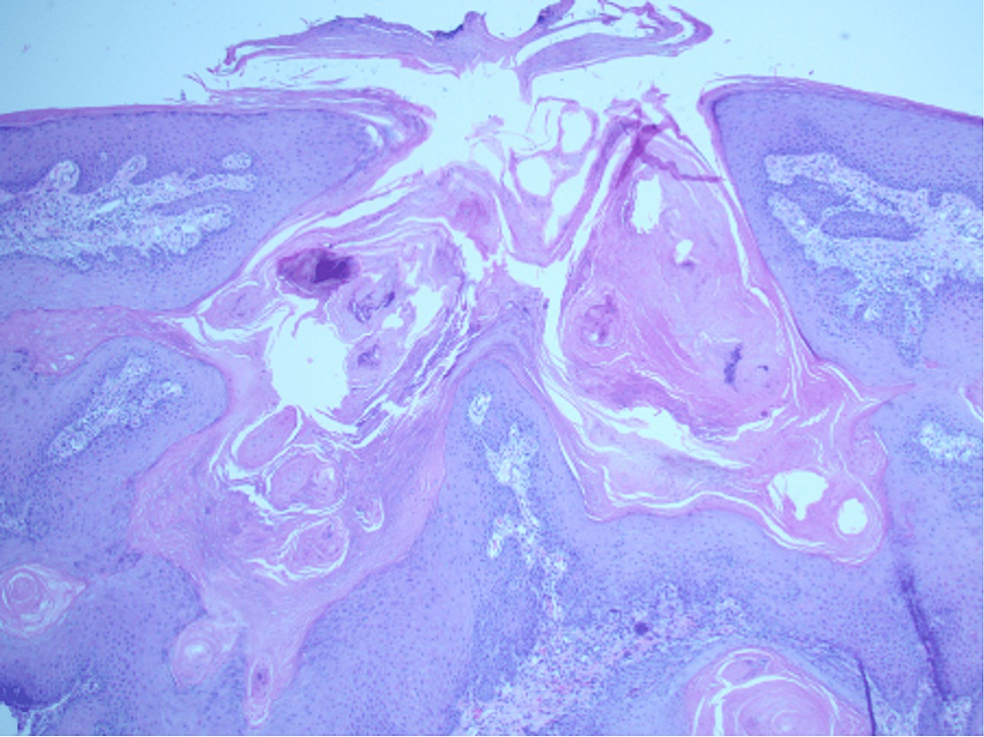
Shave biopsy of the proximal right anterior lower leg confirming keratoacanthoma, extending to the deep margin Sections of skin show an exo/endophytic crateriform sharply circumscribed hyperkeratotic lesion. There are bulky down growths of squamous cells extending from the base of this lesion into the underlying dermis. These contain enlarged keratinocytes with eosinophilic glassy appearing cytoplasm. This lesion extends to the deep margin of the specimen.

The lesions on her lower extremities were given a clinical diagnosis of eruptive keratoacanthomas secondary to pembrolizumab and a shave biopsy of her right anterior lower leg lesion was performed. The biopsy demonstrated features of keratoacanthoma; a crateriform hyperkeratotic lesion with eosinophilic glassy keratinocytes (Figure 4).

**Figure 5:**
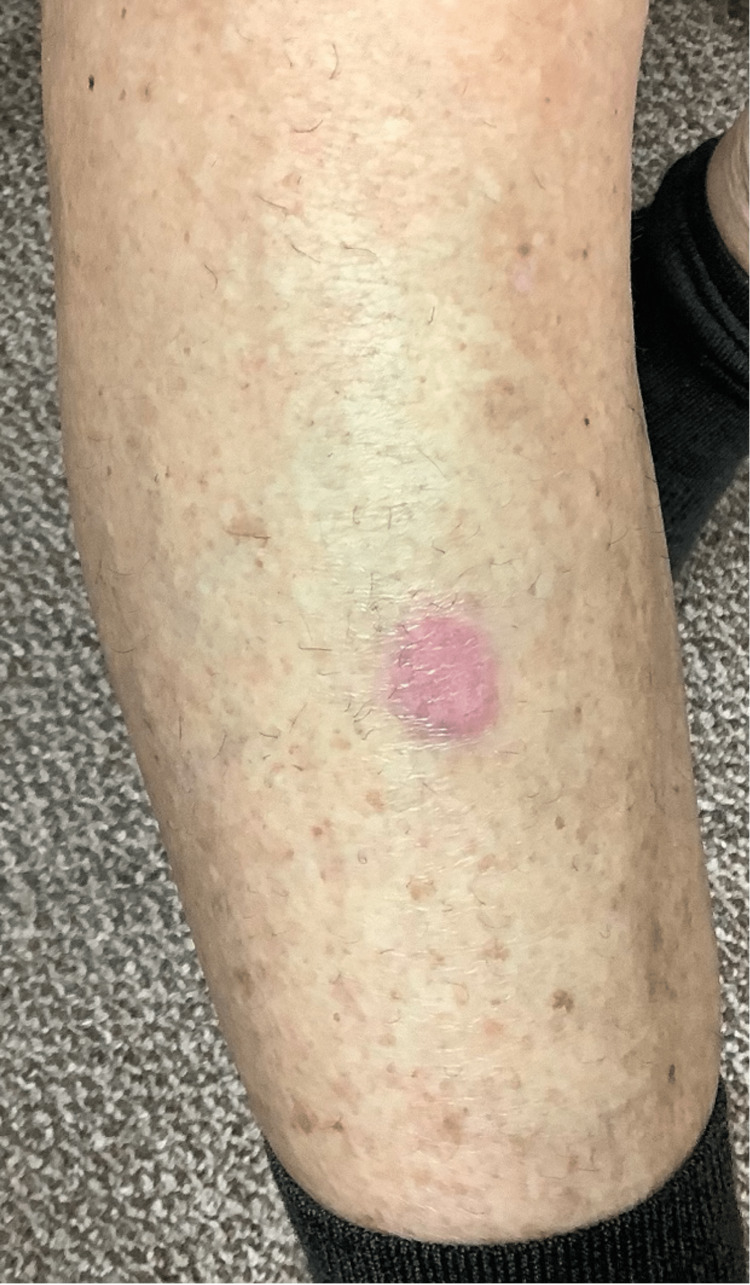
Right anterior lower leg keratoacanthoma after treatment with two intralesional injections of methotrexate

At her next visit, she received a 0.5 mL intralesional injection of methotrexate sodium 25 mg/mL solution into the biopsy-proven keratoacanthoma on her right leg. One week later, she received an additional 0.5 mL methotrexate sodium 25 mg/mL injection. The smaller keratoacanthomas were treated the same way. The patient tolerated treatment well and without adverse effects. At her three-week follow-up appointment after treatment initiation, the keratoacanthomas resolved (Figure 5), required no further treatment, and have not recurred since. The patient continued to receive pembrolizumab infusions after the diagnosis of eruptive keratoacanthomas. At her six-month follow-up appointment, the keratoacanthomas had not recurred and the patient reported decreased pruritus and flaring of the lichenoid drug eruption on her back. 

## Discussion

Keratoacanthomas are cutaneous lesions that often present as crateriform nodules. Single lesions are most common and may regress spontaneously [4]. Eruptive keratoacanthomas are multiple lesions that appear within a short timeframe. Drug-induced lichen planus has been reported as a rare dAE, which may develop after treatment with anti-PD-1 drugs [5,6]. In these reactions, keratinocytes that express PD-ligand 1 (L1) are affected, resulting in infiltration of the basal membrane and sub-epithelium by CD4/CD8 positive lymphocytes and keratinocyte death [7].

In this case, the patient undergoing pembrolizumab immunotherapy developed purple to violaceous, polygonal, papules and plaques on her upper back consistent with lichen planus and multiple hyperkeratotic papules on her lower extremities skin consistent with keratoacanthoma on biopsy. The most likely provoking factor explaining the development of our patient's lichen planus and eruptive keratoacanthomas is the inflammatory response caused by the anti-PD-1 immunotherapy drug, pembrolizumab. The complete resolution of her keratoacanthomas after treatment with intralesional methotrexate further supports that this may represent an immune-related dAE.

## Conclusions

This report demonstrates a unique case of concurrent lichen planus and eruptive keratoacanthomas, caused by immunotherapy for lung cancer. Although several therapies have been proposed, the results of this case align with findings in current literature that pembrolizumab-induced lichen planus and keratoacanthomas can be controlled with the conservative management of topical corticosteroids and intralesional methotrexate, respectively, allowing patients to continue pembrolizumab therapy. More research is needed to further describe the mechanism behind pembrolizumab-induced dAEs.
